# Impact of COVID-19 on Radiation Oncology, an Austrian Experience

**DOI:** 10.3390/curroncol28060404

**Published:** 2021-11-17

**Authors:** Julian Mangesius, Christoph Reinhold Arnold, Thomas Seppi, Stephanie Mangesius, Mario Brüggl, Paul Eichberger, Ute Ganswindt

**Affiliations:** 1 Department of Radiation Oncology, Medical University of Innsbruck, 6020 Innsbruck, Austria; ch.arnold@protonmail.com (C.R.A.); thomas.seppi@i-med.ac.at (T.S.); mario.brueggl@tirol-kliniken.at (M.B.); paul.eichberger@tirol-kliniken.at (P.E.); ute.ganswindt@i-med.ac.at (U.G.); 2 Department of Neuroradiology, Medical University of Innsbruck, 6020 Innsbruck, Austria; stephanie.mangesius@i-med.ac.at

**Keywords:** COVID-19, SARS-CoV-2, cancer, radiotherapy

## Abstract

The COVID-19 pandemic has an unprecedented impact on cancer treatment worldwide. We aimed to evaluate the effects of the pandemic on the radiation treatment of patients in order to provide data for future management of such crises. We compared the number of performed radiotherapy sessions of the pandemic period from February 2020 until May 2021 with those of 2018 and 2019 for reference. At our department, no referred patients had to be rejected or postponed, nor any significant changes in fractionation schedules implemented. Nevertheless, there was a substantial drop in overall radiotherapy sessions in 2020 following the first incidence wave of up to −25% (in June) in comparison to previous years. For breast cancer, a maximum decline of sessions of −45% (July) was recorded. Only a short drop of prostate cancer sessions (max −35%, May) followed by a rebound (+42%, July) was observed. Over the investigated period, a loss of 4.4% of expected patients never recovered. The severe impact of COVID-19 on cancer treatment, likely caused by retarded diagnosis and delayed interdisciplinary co-treatment, is reflected in a lower count of radiotherapy sessions. Radiation oncology is a crucial cornerstone in upholding both curative treatment options and treatment capacity during a pandemic.

## 1. Introduction

Unprecedented in modern medicine, the SARS-CoV-2 pandemic had, and still has, a detrimental impact on social life, economy, and health care systems around the globe. In order to mitigate the ramifications of the pandemic, various measures have been implemented worldwide, such as face masks mandates, mass quarantines, and travel restrictions.

Cancer patients represent a primary risk group, since they are generally of older age, afflicted by comorbidities, and an often-compromised immune system. Therefore, they are at higher risk of COVID-19 mortality [1,2,3,4,5]. For these patients, timely diagnosis and immediate initiation of treatment is of utmost importance to ensure optimal outcome. A major challenge in the care for cancer patients during the pandemic is finding an acceptable balance between protecting them from infection during clinical routine, and not delaying diagnosis and subsequent therapy. In evidence, there is abundant data on the devastating effects of delayed cancer diagnoses, with patients presenting at more advanced stages, ultimately leading to excess cancer mortality [6,7,8,9].

Radiation oncology departments face a unique challenge during this pandemic, because the majority of patients attend these institutions on a daily basis for a period of up to 8 weeks. Each stay increases the risk of being exposed to healthcare professionals and other patients, who may be asymptomatic or not yet identified carriers of the virus. Various countermeasures have been recommended by radiation oncology societies in order to warrant uninterrupted and undelayed treatment, while simultaneously reducing the risk of patients being infected during the course of their therapy [10,11,12]. Beside commonly recommended protective measures to prevent COVID-19 infections at cancer treatment centres, in the case of severe pandemic-related capacity limitations, guidelines also include an intensified use of hypofractionated treatment schedules, and a stricter evaluation of the risk/benefit ratio in each individual case, thereby giving preference to uphold primarily curative treatment [10,13,14,15,16,17].

In Austria, first COVID-19 cases were reported in February 2020 [18,19]. As a result, the Austrian government has ordered a nationwide lockdown, which came into effect on the 10th of March. Lockdown measures were incrementally lifted starting from April 14th. In autumn, the number of COVID-19 cases in Austria was rising again, thereby reaching highest infection rates recorded worldwide for a couple of weeks. This led to an accentuation of the measures, which then ultimately culminated in a second lockdown on the 3rd of November. Finally, third and fourth confluent waves at elevated infection rates had to be faced during a period lasting from January to May 2021.

In the present study, we analysed the impact of the COVID-19 pandemic and resulting governmental measures on radioncologic care. Our radiation oncology department is the only institution in the North Tyrolean area.

## 2. Materials and Methods

In this retrospective single-centre study, we analysed the number of daily-performed radiation therapy sessions treated at the Department of Therapeutic Radiology and Oncology of the Medical University of Innsbruck. We obtained data for all external beam radiation therapy (RT) sessions performed in the pre-pandemic years of 2018 and 2019 and compared it to pandemic period of 16 months from February 2020 until May 2021.

All primary tumour entities were included. In addition to the analysis of total session count, the three most frequent malignancies (26.9% breast, 28.9% prostate, and 10.4% lung cancer in our cohort) were analysed separately to exemplarily depict tumour specific changes in treatment counts during the pandemic. Included patients received curative (78.6%) as well as palliative (21.4%) treatments. Non-oncologic RT sessions were excluded from this analysis. Datasets extracted for use in this study included daily distributions of primary cancer types (defined according to ICD-10).

The progressively increased application of hypofractionation during the last years has been considered by monthly correction over the entire reference and the pandemic period. All reported results have thereby been normalised to eliminate any decrease of session count per patient and month, which cannot be traced back to the pandemic impact.

For statistical analysis and graphical presentation, SPSS statistics 26 software (IBM Cooperation, Armonk, NY, USA) was used. The mean number of RT sessions performed per day were pooled and analysed by month. We calculated the absolute and relative monthly difference during the pandemic compared to the mean session count of the previous two years. Normality distribution of data was assessed using the Kolmogorov-Smirnov test. For comparison, one sample *t*-test was performed for each month. The same analytic procedure was performed for mean daily treatment sessions for breast, prostate, and lung cancer. The level of significance was set to *p* < 0.05.

## 3. Results

Overall, an average of 2577 single RT sessions were performed per month during 16 months of the pandemic (February 2020 to May 2021). In comparison, a monthly average of 2701 RT sessions was performed in the reference months of the previous two years (January 2018 to May 2019). This corresponds to a cumulative loss of −4.41% RT sessions in the pandemic period (41,312 pandemic vs. 43,216 pre-pandemic sessions). These constitute 1471 whole RT treatment courses during the 16 months of pandemic compared to 1539 in the reference period (corresponding to a cumulative loss of 69 patients).

**Figure 1:**
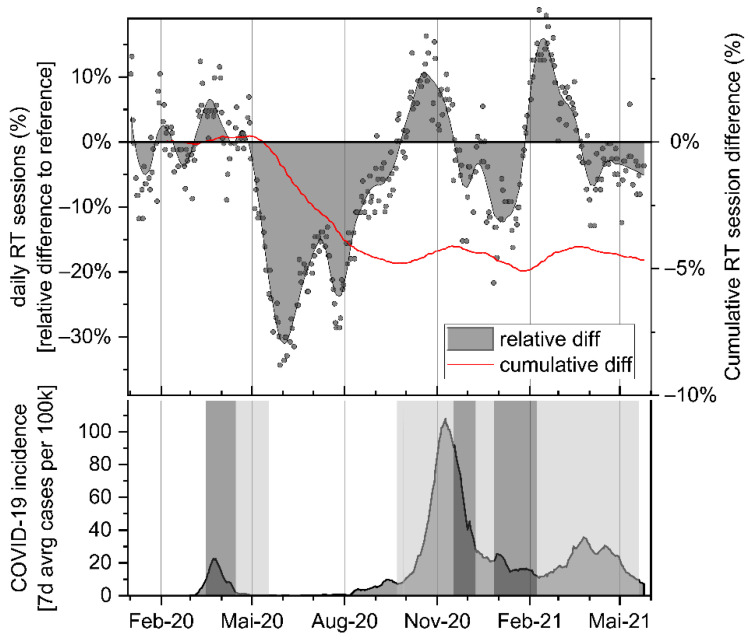
Upper panel: relative and cumulative difference of RT sessions performed during the pandemic period in comparison to the reference period of 2018 and 2019. Area plot: daily relative difference. Red line: Cumulative lost RT courses. COVID-19 incidence (7-day average count of confirmed new cases per 100 k people). Shaded areas indicate strict lockdown (dark grey) and moderate (light grey) national restrictions.

Comparing the mean daily percentage of session difference between pandemic- and pre-pandemic months, statistically significant monthly drops can be observed in the period from May to August 2020 as a consequence of the first incidence wave (−18.9%, −24.9%, −19.5%, −11.6%; *p* < 0.001; see Table 1 and Figure 1). From July onward, session counts slowly normalized to normal levels, finally surpassing these by October 2020 (+6.9%; *p* = 0.003). Meanwhile, COVID-19 infection incidences were escalating to highest levels, reversing the rebound in the following month of November, and leading to an additional minimum in sessions counts in January 2021 (−7.4%, *p* < 0.001). Between the confluent third and fourth pandemic waves at constantly elevated infection rates, a short rebound in RT session count was observed in February 2021 (+13.4%; *p* < 0.001), again followed by a less pronounced drop until of the observed pandemic period in May 2021 (−4.5%; *p* = 0.001). No significant fluctuations in performed palliative treatment sessions were recorded during the course of the observed pandemic months.

The cumulative count of pandemic-related patient loss showed a first low point in September ′20 (−4.13%; corresponding to 64 lost patients out of 1539 expected in the assessed period of 16 months), and a second cumulative minimum in January 2021 (−4.87%, 75 lost patients). At the end of the investigated pandemic period in May ′21, a cumulative net loss of 69 out of expected 1539 patients (−4.4%) was recorded at our department (Table 2 and Figure 1).

**Figure 2:**
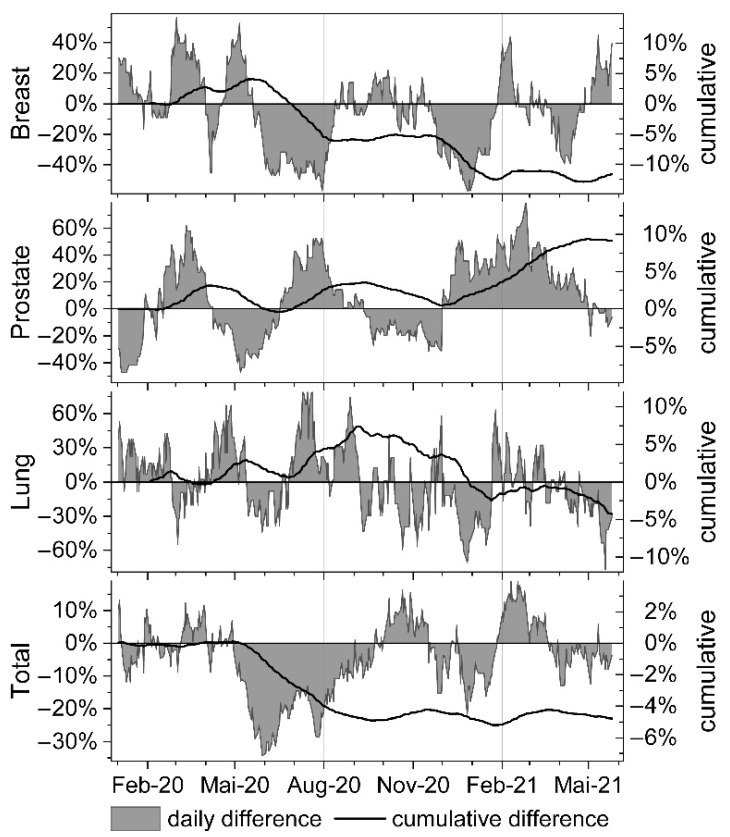
Relative and cumulative difference of total, as well as of breast, prostate and lung cancer-related RT sessions performed during the pandemic period in comparison to the reference months of 2018 and 2019. Area plot: daily relative difference.

Of the three separately investigated tumour entities (Table 3 and Figure 2), the drops in breast cancer treatments (accounting for 27.1% of all treated patients) were most pronounced following the first and second pandemic waves (−39.2% in June and −45.3% in July ′20; 40.0% in December ′20 and 21.2% in January ′21). At the end of the assessed pandemic period, a cumulative net loss of 11.7% of breast cancer patients was recorded (Table 2 and Figure 2).

Conversely, RT sessions attributed to prostate cancer patients (accounting for 27.6% of all treated patients) showed a short pronounced decline in May (−35.2%) followed by a rebound in July ′20 (+41.8%). During the second wave in October and November ′20 (−18.7%; −24.2%), a novel minimum was observed immediately followed by a second rebound lasting from December ′20 to April ′21 (highest in Feb ′21: +54.6%). Cumulatively, a net surplus of 8.7% of prostate cancer patients were treated in the pandemic period when compared to the reference period in 2018/2019 (Table 2 and Figure 2).

Lung cancer treatments (accounting for 11.4% of all treated patients), showed periodic fluctuations with most pronounced minima during October ′20 until January ′21 (Table 3), resulting in a cumulative net loss of patients of −6.5% (Table 2 and Figure 2).

## 4. Discussion

Maintaining high-quality cancer care also during a pandemic is of paramount importance, as it is known that delays or interruptions of diagnosis or treatment adversely affects prognosis. In this study, we evaluated the course of the number of daily radiation treatment sessions throughout 16 consecutive months of pandemic, and compared it to 16 corresponding pre-pandemic reference months. Thereby, we wanted to assess the impact of the COVID-19 pandemic, and of the subsequently enacted countermeasures on cancer care in the Austrian federal province of Tyrol including some adjacent referrer districts (population ~1 mio.).

Most oncologic therapies are multimodal treatment concepts involving surgery, systemic therapies, and radiotherapy. Disruptions of any link in the chain of diagnosis and therapy will affect subsequent treatments and may impair patient outcome. Since radiation therapy is usually not the initial modality in a cancer patient’s treatment course, entering RT is highly dependent on the timely completion of previous diagnostic procedures, as well as of surgical interventions or systemic therapies. As such, radiation oncology departments are disproportionally affected by disruptions in the course of interdisciplinary oncologic care. Consequently, reduced numbers of radiation treatment sessions are clearly monitoring any obstacle blocking the preceding treatment progression.

The impact of the pandemic on timely diagnosis and primary treatment of cancer patients is evident according to multiple reports around the world [8,20,21,22]. Early modelling from the UK, suggested that delays in diagnosis of only four cancer types, namely breast, colorectal, esophageal, and lung cancer, may lead to an increase of cancer-related deaths ranging from 4.8 to 16.6% five years after diagnosis [8].

Importantly, at our radiation oncology department, no referrals have been rejected nor have any treatments been delayed during the observed 16 months of pandemic. Many practice recommendations for radiotherapy during a pandemic [10,13,14,15] discussed an increased use of hypofractionation as means for minimizing the risk of viral transmission, by reducing contact frequency and duration of treatment sessions. By nature and if indicated, hypofractionation can alleviate strain on both human resources and equipment. In fact, use of hypofractionation has increased also at our department over the last years, which resulted in an overall lower session count per patient in 2020. However, the proportion of hypofractionated schedules largely remained constant if compared to the pre-lockdown reference months. The initially observed post-lockdown drop in treatment sessions was therefore not attributable to altered treatment schedules with further increased application of hypofractionation. Finally, hypofractionation as an emergency measure to counteract workflow limitations during the COVID-19 pandemic, as reported by other clinics all over the world, was never indicated at our department. Maintenance of full treatment capacity at our department is primarily attributable to the rapidly implemented safety measures, which proofed to be efficient, and up to now successfully prevented any significant outbreak of COVID-19 at our radiation oncology department.

Similar to our experience, also other departments of radiation oncology managed to maintain quality and capacity of care by implementing protective measures. Such measures include but are not limited to rearranging staff in small working units, facilitating home office, wearing of personal protective equipment by both patients and staff, routinely taking temperature, postponement of treatment of benign diseases, weekly antigen testing, spatial and temporal separation of patients, avoiding in-person meetings through teleconferences, and by implementing follow-up visits by telephone. By strictly following these measures in accordance with published recommendations [10,13,14,16,17], COVID-19 outbreaks as well as potentially detrimental changes in treatment regimens and patient flow can be avoided. During the entire pandemic period, our department has continued normal routine in radiation treatments without incurring interruptions or limitations potentially caused by the promptly implemented epidemiological safety measures. We were not forced to implement any significant change to our regular treatment regimens. No indicated treatment of oncologic patients by radiation therapy has been cancelled or postponed because of the pandemic.

However, the pandemic is known to have caused severe disruptions in diagnosis and therapy [8,21,23] prior to RT. This is attributable to patients avoiding or postponing contact with healthcare providers as well as to an observed reduction in the capacity of routine cancer screening following governmental lock-down measures. Additionally, surgical interventions have been postponed to maintain sufficient ICU capacity especially during the first wave of the pandemic in spring ′20, meanwhile bridging eligible patients with neoadjuvant endocrine therapy [24]. Lastly, also COVID-19 cases among hospital staff have led to impairments in timely administration of cancer therapies. The observed overall drop in RT sessions at our department is therefore a direct consequence of missing or delayed referral of patients, which began two months after the first rise of COVID-19 cases during the initial phase of the pandemic.

Analysis of mean daily treatment sessions during 16 months of pandemic revealed a marked downturn of up to 24.9% from May to August ′20 following the first lockdown, which lasted from March 10th to April 14th. This equates to a total of 2630 missing therapy sessions within these four months. Data by Spencer et al. corroborate these findings by a decrease in RT attendances of up to 31.5% observed within the months of April to June ′20 [25]. The recorded cumulative net loss of cancer patients at our department (−4.4%) during the pandemic until end of May ′21 indicates that a significant number of patients permanently missed the indicated cancer treatment. This will likely translate to excess cancer-related deaths in the future [8].

In detail, our data show breast cancer treatment to be the most affected, with two significant sudden patient drops of up to −45.3% incurred in the months of June and July ′20 as well as in December ′20 and January ′21. The observed two-step decline correlates very well with a two-month delay-response following the pandemic incidence peaks of the first and second wave, and with subsequent lock-down measures of the government (Figure 1 and Figure 2). Consequently, cumulative net loss of breast cancer patients after the first wave corresponds to −5.2% (from February until August ′20 ) and following the second wave to −10.3% (until December ′20). The confluent third and fourth pandemic waves in spring ′21 contributed only moderately to the finally observed cumulative net loss of −11.9% breast cancer patients in May ′21. Contrary to the observed rebound effect recorded after preceding declines in prostate cancer patients after the first and second wave of the pandemic (Figure 2), no net recovery in treatment counts of breast cancer patients was detected until May ′21. Therefore, it is unlikely that a delay in the indicated sequence of oncologic therapies only caused by transiently postponed surgical interventions during April and May ′20 might represent a reasonable explanation for this significant patient loss. Besides this, if temporary capacity restrictions in surgery were the leading cause for the observed patient drops, one would expect that the majority of these patients would have (re-)entered the treatment course at least within one year after the first wave. However, this was not the case, and even though some of the apparently lost patients might present themselves for radiotherapy in the future, the incurred delay in treatment-start will exert its negative effects on prognosis and subsequent treatment options. In conclusion, other factors than postponed surgical interventions, such as delayed screening and diagnosis, as a consequence of either reduced screening capacity or patients less contacting health care providers because fearing COVID-19 infection, have to be taken into account, as also reported by others [9]. Therefore, special attention will be dedicated to the next months with regard to potentially recovering breast cancer treatment counts.

Interestingly, if compared to the average count of previous years, only brief reductions in monthly treatments have been recorded for prostate cancer patients following the first and second pandemic waves (−35.2% in May ′20, and −24.2% in November ′20). However, the short drops both were fully compensated by rebounds during subsequent pandemic months. In addition, the cumulated net count of treated prostate cancer patients at our department revealed a surplus of +3.0% within the first 12 months of pandemic if compared to reference months of the previous two years. This unexpected increase in treated patients may be attributable to the fact that radiation therapy increasingly represents the primary treatment for prostate cancer. In addition, this schedule is less vulnerable to COVID-19-related delays attributable to other disciplines. It is also feasible that a potential drop in screening and diagnosis may be compensated for by a continuously ongoing shift from surgical intervention to radiation treatment. Regarding prostate cancer patients, this assumption is likely to be confirmed by the cumulative RT session count after 16 months of pandemic exceeding the pre-pandemic reference level by +8.7%.

Radiotherapy of lung cancer patients was not significantly affected by the first pandemic wave. In fact, a significant cumulative net loss of patients was only observed after November ′20, as a consequence of the second, and most intense wave. During the confluent third and fourth waves, patient counts continuously declined to a moderate total loss of −6.5% until May ′21. However, it is not clear whether this decline in treatment courses is fully attributable to pandemic effects, since number of lung cancer cases treated is about one third of breast and prostate cancer. In part, the decline in radiotherapy sessions of lung cancer patients may also follow normal fluctuations of incidence rates and overlapping seasonal differences.

Understanding the full implications of the COVID-19 pandemic on oncologic care is complex. This work will contribute to illuminate it’s impacts on patients requiring radiotherapy, and on the scope of action at disposition to providers of radiotherapy. Our department is the sole provider of radiation therapy for the entire region of Tyrol, Austria, and there is no mentionable transfer of oncologic patients to other states in Austria nor internationally. Therefore, our data represents an accurate portrayal of radioncologic care in this region. While the reported drop in treatment numbers and available epidemiologic modelling can provide an estimation of the future impact on oncologic outcome and excess cancer-related deaths, we unfortunately do not have accurate staging information available yet. However, a mean shift to a more advanced stage at treatment is likely to be observed in the coming months. Furthermore, while our data is in good agreement with published experience around the world, the impact of COVID-19 on oncologic care is highly dependent on local case numbers, effectiveness of official guidelines and restrictions, the robustness of the national health care system, and the effective coordination oncologic disciplines.

## 5. Conclusions

The COVID-19 pandemic has proven disruptive for oncologic care, the ramifications of which will become evident in the years to come. Its unavoidable impact on interdisciplinary oncologic care is finally reflected by a reduced number of RT treatment courses. In evidence, the bottlenecks might be attributable to postponed surgical interventions to provide sufficient ICU capacity for COVID-19 patients, as well as to temporary cessation of routine screenings for early cancer detection. Our data suggests that breast cancer care is disproportionally affected by the current pandemic, whereas transient limitations in surgical treatment of prostate cancer can be compensated by further increased utilisation of radiation therapy. Since no recovery of the 4.4% cumulatively lost patients was observed within 12 months following the first pandemic wave, excess cancer-related deaths have to be expected over the next years. Full curative radio-therapeutic options for all cancer patients can be upheld even during a pandemic by adhering to strict protection measures.

## Figures and Tables

**Table 1:** Relative difference of daily RT session counts during the COVID-19 epidemic in comparison to the corresponding pre-pandemic reference period.

Month	N Days	Mean Session Difference [%]	*p*-Value	CI 95% Lower	CI 95% Upper
February 20	20	−1.94%	0.133	−4.53%	0.65%
March 20	22	4.11%	0.001	1.88%	6.33%
April 20	21	0.54%	0.491	−1.07%	2.16%
May 20	19	−18.93%	<0.001	−23.06%	−14.79%
June 20	20	−24.92%	<0.001	−27.38%	−22.45%
July 20	23	−19.48%	<0.001	−21.72%	−17.24%
August 20	21	−11.64%	<0.001	−13.39%	−9.88%
September 20	22	−3.53%	0.001	−5.39%	−1.66%
October 20	21	6.90%	0.003	2.64%	11.15%
November 20	21	−1.63%	0.578	−7.63%	4.38%
December 20	21	−6.75%	<0.001	−9.82%	−3.67%
January 21	20	−7.38%	<0.001	−9.98%	−4.78%
February 21	20	13.40%	<0.001	11.81%	14.98%
March 21	23	2.78%	0.008	0.80%	4.76%
April 21	21	−4.59%	<0.001	−6.30%	−2.87%
May 21	15	−4.36%	0.001	−6.55%	−2.17%

**Table 2:** Loss of expected patients during the pandemic in comparison to the reference period. Percentage values depict the relative cumulative difference to the total expected number of treatment courses observed during 16 months of the progressing pandemic period (pre-pandemic reference period: February 2018 until May 2019).

Month	Breast	Prostate	Lung	Total
February 20	−0.34%	0.54%	−0.08%	−0.11%
March 20	1.12%	3.01%	−1.68%	0.26%
April 20	1.14%	2.49%	−0.21%	0.45%
May 20	1.21%	−0.59%	−1.03%	−1.06%
June 20	−1.75%	−0.57%	−1.59%	−2.61%
July 20	−4.81%	2.53%	1.21%	−3.42%
August 20	−5.20%	3.02%	2.25%	−4.27%
September 20	−4.43%	3.11%	1.76%	−4.09%
October 20	−4.80%	1.33%	0.23%	−4.10%
November 20	−5.00%	0.04%	−0.42%	−3.89%
December 20	−7.80%	1.95%	−2.69%	−4.07%
January 21	−10.34%	2.99%	−4.34%	−5.01%
February 21	−9.98%	5.38%	−4.77%	−4.26%
March 21	−10.27%	8.13%	−5.06%	−3.63%
April 21	−11.30%	9.24%	−6.09%	−3.76%
**May 21**	**−11.65%**	**8.66%**	**−6.53%**	**−4.41%**

The bold emphasizes that this row represents the cumulative numbers after 16 months, which are referenced in the text.

**Table 3:** Relative difference of breast, prostate and lung cancer RT session count during the COVID-19 epidemic in comparison to the pre-pandemic reference period.

Month	Relative Difference in Daily Session Counts [%]					
	Breast		Prostate		Lung	
	Mean	95% CI	Mean	95% CI	Mean	95% CI
February 20	2.51%	−4.72%/9.75%	15.08%	5.27%/24.89% **	13.49%	5.4%/21.58% **
March 20	32.12%	26.9%/37.34% **	36.69%	30.52%/42.85% **	−14.28%	−21.18%/−7.37% **
April 20	1.89%	−9.83%/13.6%	−8.12%	−13.05%/−3.18% **	31.32%	22.01%/40.63% **
May 20	12.90%	1.79%/24.02% *	−35.21%	−37.87%/−32.54% **	−7.15%	−18.21%/3.92%
June 20	−39.23%	−41.87%/−36.59% **	−0.88%	−7.51%/5.75%	−13.60%	−22.06%/−5.14% **
July 20	−45.33%	−47.61%/−43.06% **	41.76%	38.14%/45.38% **	42.14%	31.71%/52.57% **
August 20	−5.44%	−13.1%/2.23%	13.00%	8.58%/17.43% **	28.16%	17.98%/38.34% **
September 20	3.45%	−0.73%/7.63%	−5.86%	−11.03%/−0.69% *	−6.68%	−18.63%/5.26%
October 20	0.94%	−6.01%/7.89%	−18.68%	−23.78%/−13.58% **	−13.05%	−25.37%/−0.73% *
November 20	−8.17%	−16.76%/0.41%	−24.24%	−30.88%/−17.6% **	−15.09%	−28.32%/−1.85% *
December 20	−39.98%	−44.79%/−35.18% **	28.68%	21.5%/35.86% **	−31.57%	−41.52%/−21.62% **
January 21	−21.23%	−29.5%/−12.97% **	36.61%	32.67%/40.54% **	−13.12%	−30.33%/4.1%
February 21	15.40%	6.83%/23.96% **	54.61%	47.75%/61.48% **	7.57%	−0.94%/16.08%
March 21	−4.26%	−8.69%/0.16%	34.99%	29.94%/40.05% **	−0.14%	−8.05%/7.77%
April 21	−17.30%	−24.45%/−10.16% **	17.03%	13.5%/20.57% **	−13.38%	−19.5%/−7.26% **
May 21	22.39%	15.94%/28.84% **	−4.48%	−8.17%/−0.79% *	−34.39%	−44.28%/−24.49% **

* one sample *t*-test *p* < 0.05. ** one sample *t*-test *p* < 0.01.

## Data Availability

The data presented in this study are available on request from the corresponding author.
